# Draft Genome of *Spiribacter salinus* M19-40, an Abundant Gammaproteobacterium in Aquatic Hypersaline Environments

**DOI:** 10.1128/genomeA.00179-12

**Published:** 2013-02-14

**Authors:** Maria Jose Leon, Rohit Ghai, Ana Beatriz Fernandez, Cristina Sanchez-Porro, Francisco Rodriguez-Valera, Antonio Ventosa

**Affiliations:** a Department of Microbiology and Parasitology, Faculty of Pharmacy, University of Sevilla, Sevilla, Spain; b Evolutionary Genomics Group, Departamento de Producción Vegetal y Microbiologia, Universidad Miguel Hernández, San Juan de Alicante, Alicante, Spain

## Abstract

We have previously used a *de novo* metagenomic assembly approach to describe the presence of an abundant gammaproteobacterium comprising nearly 15% of the microbial community in an intermediate salinity solar saltern pond. We have obtained this microbe in pure culture and describe the genome sequencing of the halophilic photoheterotrophic microbe, *Spiribacter salinus* M19-40.

## GENOME ANNOUNCEMENT

Hypersaline systems, e.g., solar salterns, are extreme environments of salinity higher than that of seawater. Microbes adapt to high salinities using two strategies. In the salt-out strategy, cells accumulate compatible solutes (e.g., ectoine and betaine) in the cytoplasm to exclude inorganic salts (mostly Na^+^). However, in the salt-in strategy, they actively maintain a high internal ionic concentration of potassium ions and have a significantly higher proportion of positively charged amino acids (arginine and lysine) in their proteins to maintain solubility. A range of metagenomic data sets from different salinities has been analyzed previously, and novel microbes were identified using a *de novo* metagenomic assembly approach (1). In that previous work, in a metagenomic data set from a solar saltern pond of 19% salinity, metagenomic assembly yielded 15 contigs (a total of 94 kb; G+C content, 63% to 67%) that could clearly be attributed to a gammaproteobacterium related to the genera *Alkalilimnicola* and *Nitrococcus*. However, due to the lack of a complete genome, not many observations could be made, except that the isoelectric points of the predicted proteins suggested that the microbe uses a salt-out strategy.

In an effort to culture these novel microbes, we undertook further sampling and culturing from the same saltern (Santa Pola, Spain) and from another with similar salinity (Isla Cristina, Spain), using 16S rRNA PCR for colony identification. A slow growing microbe, with a 16S rRNA sequence related to *Alkalilimnicola*, was obtained from the Isla Cristina saltern. In liquid culture, the cells appeared as spiral rods and clustered into aggregates, likely due to polysaccharide production. On solid medium, the cells appeared as slender small rods. We have named this microbe *Spiribacter salinus* M19-40, and a formal taxonomic description is in progress.

Whole-genome shotgun (WGS) sequencing of *S. salinus* M19-40 was performed using Illumina HiSeq 2× 100-bp paired-end (PE) reads and Pacific Biosciences 3- to 5-kb reads. Illumina reads at 100× coverage and long PacBio error-corrected reads (2) at a coverage of ~50× were assembled using MIRA (3) into 6 contigs (N_50_, 1,369,469). The total genome size was 1.74 Mb (1,742,247 bp), with a G+C content of 62.65%. We identified 1,706 protein-coding genes (4) and a single rRNA operon (5). Forty-five tRNAs for all canonical 20 amino acids were identified (6). Protein-coding genes were compared to NCBI nonredundant (NR) and Kyoto Encyclopedia of Genes and Genomes (KEGG) databases for functional annotation.

Analysis of the global predicted proteome pI, and the presence of ectoine biosynthesis genes, confirm that *S. salinus* appears to have a salt-out strategy. No flagellar genes could be found, indicating the absence of swimming motility. The presence of a rhodopsin gene indicated that *S. salinus* is probably a photoheterotroph. The most similar 16S rRNA gene sequences in the Ribosomal Database Project (7) appeared to be uncultured *Ectothiorhodospiraceae* bacteria and *Alkalilimnicola* species. The identity similarity of the single 16S rRNA gene to that of the *Alkalilimnicola* 16S rRNA gene was 95%. However, the average nucleotide identity between the genomes was only 67.92%, which is indicative of the microbe belonging to a new genus.

### Nucleotide sequence accession numbers.

This Whole Genome Shotgun project has been deposited at DDBJ/EMBL/GenBank under the accession no. ANKZ00000000. The version described in this article is the first version, ANKZ01000000.

## References

[1] Ghai R Pašíc L Fernández AB Martin-Cuadrado AB Mizuno CM McMahon KD Papke RT Stepanauskas R Rodriguez-Brito B Rohwer F Sánchez-Porro C Ventosa A Rodríguez-Valera F 2011 New abundant microbial groups in aquatic hypersaline environments. Sci. Rep. 1:135 22355652 10.1038/srep00135 PMC3216616

[2] Koren S Schatz MC Walenz BP Martin J Howard JT Ganapathy G Wang Z Rasko DA McCombie WR Jarvis ED Phillippy AM 2012 Hybrid error correction and *de novo* assembly of single-molecule sequencing reads. Nat. Biotechnol. 30:693–700 22750884 10.1038/nbt.2280 PMC3707490

[3] Chevreux B 2005 MIRA: an automated genome and EST assembler, p 45–46 Computer science and biology: proceedings of the German conference on bioinformatics (GCB)99. Ruprecht-Karls University, Heidelberg, Germany

[4] Hyatt D Chen GL Locascio PF Land ML Larimer FW Hauser LJ 2010 Prodigal: prokaryotic gene recognition and translation initiation site identification. BMC Bioinformatics 11:119 20211023 10.1186/1471-2105-11-119 PMC2848648

[5] Lagesen K Hallin P Rødland EA Staerfeldt HH Rognes T Ussery DW 2007 RNAmmer: consistent and rapid annotation of ribosomal RNA genes. Nucleic Acids Res. 35:3100–3108 17452365 10.1093/nar/gkm160 PMC1888812

[6] Lowe TM Eddy SR 1997 tRNAscan-SE: a program for improved detection of transfer RNA genes in genomic sequence. Nucleic Acids Res. 25:955–964 9023104 10.1093/nar/25.5.955 PMC146525

[7] Cole JR Wang Q Cardenas E Fish J Chai B Farris RJ Kulam-Syed-Mohideen AS McGarrell DM Marsh T Garrity GM Tiedje JM 2009 The Ribosomal Database Project: improved alignments and new tools for rRNA analysis. Nucleic Acids Res. 37:D141–D145 19004872 10.1093/nar/gkn879 PMC2686447

